# A Canadian algorithm for upper gastrointestinal cancer management

**DOI:** 10.3389/fonc.2025.1548637

**Published:** 2025-04-14

**Authors:** Frédéric Lemay, Amindeep S. Sandhu, Barry D. Stein, Rachel Goodwin

**Affiliations:** ^1^ Division of Gastroenterology, Department of Medicine, Université de Sherbrooke, Sherbrooke, QC, Canada; ^2^ Schulich School of Medicine and Dentistry, Western University, London, ON, Canada; ^3^ Colorectal Cancer Canada, Montréal, QC, Canada; ^4^ Division of Medical Oncology, Department of Medicine, University of Ottawa, The Ottawa Hospital Regional Cancer Centre, Ottawa, ON, Canada

**Keywords:** best practices, algorithm, esophageal, gastric, gastroesophageal junction, immunotherapy, nutrition, survivorship

## Abstract

Recent advances in immunotherapy have changed the treatment landscape for cancers of the upper gastrointestinal (GI) system. Immune checkpoint inhibitors can lead to better survival and improved quality of life for affected individuals. Adopting new treatment strategies in real-world practice can be challenging, and algorithms that are easy to implement in Canadian oncology practices would benefit clinicians and patients. In this study, we present expert opinion on best practices for upper GI cancer management, including a new algorithm that integrates the latest evidence for screening, workup, diagnosis, treatment, and survivorship. The algorithm is based on a novel approach comprising a case-based, accredited educational program with asynchronous discussion among clinicians practicing across Canada, with the input of expert medical oncologists and gastroenterologists. A needs assessment was employed to determine current areas of educational need in the field of upper GI cancers, and a patient representative provided insights into patient concerns and priorities. The best practices described here include seeking patient input throughout treatment, integrating immune checkpoint inhibitors into systemic therapy for both localized and advanced disease, and providing comprehensive supportive care throughout the treatment and survivorship journey.

## 1 Introduction

Cancers of the upper gastrointestinal (GI) system are aggressive and heterogeneous. Esophageal cancer (EC), gastroesophageal junction cancer (GEJC) and gastric cancer (GC) are often diagnosed at advanced stages, and as a result, treatment options are limited (1–3). Interdisciplinary management and comprehensive supportive care are crucial (1).

Immunotherapy has improved survival and quality of life for individuals with EC and GC (4, 5). However, treatment paradigms are still being refined (1, 2, 6, 7), and individuals who are treated at high-volume centers tend to have better outcomes (8, 9). An Ontario study reported worse survival in routine practice compared with clinical trials (10). In an American study, one-quarter of patients with advanced cancer received no treatment, and treatment was of limited duration (3). Clinicians aiming to improve outcomes would benefit from guidance on integrating novel therapies into treatment and optimizing interdisciplinary care.

Current care pathways (11–14) may not reflect the latest advances and may not apply to all regions. In this Perspective we present expert opinion on best practices, based on an educational program with 77 participants from practice settings across Canada, with input from an expert Scientific Planning Committee. These best practices include patient input and reflect the latest evidence as it applies to the Canadian health care system.

## 2 Algorithm development

The algorithm was developed through an accredited educational program supported by the Canadian Association of Gastroenterology (CAG) (15). The program provided an opportunity for discussion and consensus on best practices. The Scientific Planning Committee included experts with diverse backgrounds and practice settings who treat upper GI cancers: two gastroenterologists (one representing the CAG; FL and AS), a medical oncologist (RG), and a patient representative (BDS). The patient representative’s role was to ensure that patient perspectives were included in all stages of the program and in the final algorithm.

To assess educational needs, a survey was distributed to Canadian health care professionals who treat GI cancers. Questions addressed their needs for education related to diagnosis, treatment, supportive care, and other aspects of interest. The respondents were primarily medical oncologists and gastroenterologists, with other specialties such as pharmacists and oncology nurses also represented. The identified areas of educational need included biomarker testing, immunotherapy, recent clinical trials, and treatment algorithms.

The program was designed to address these areas. Three fictional case studies were developed. Seventy-seven participants, practicing in community and academic settings from all provinces and the Yukon Territory, provided feedback through an online discussion board. Participants’ specialties were primarily gastroenterology or medical oncology, but family physicians, pharmacists, and pathologists were also represented.

After providing input regarding the initial cases, participants submitted anonymized cases based on their experience, and five cases were chosen for further discussion. The patient cases and peer discussions were available for all participants to see. The information from the discussions was collected and organized into best practices for diagnosis, management with curative intent, and management of advanced disease. The process of collecting, analyzing, and organizing the best practices was overseen by the Scientific Planning Committee (including a CAG representative). The results were summarized in a share-back presentation, which is available online along with the rest of the program (15). Finally, the best practices were assembled into an algorithm. The participants and the CAG have access to all materials and discussions.

## 3 Treatment algorithm

### 3.1 Overview

**Figure 1:**
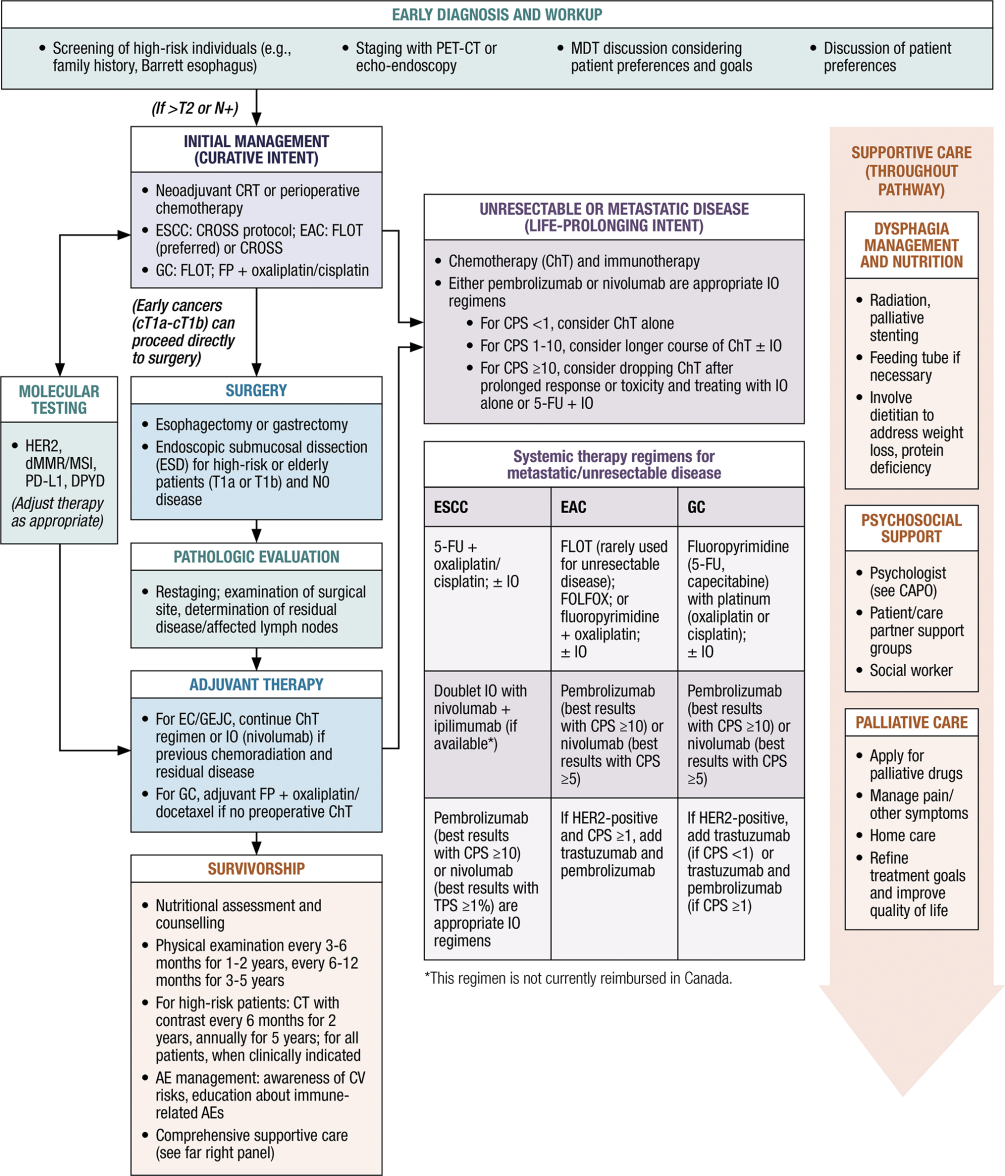
Canadian algorithm for the management of upper GI cancers. AEs, adverse events; CAPO, Canadian Association of Psychosocial Oncology; ChT, chemotherapy; CPS, combined positive score; CROSS, Chemoradiotherapy for Esophageal Cancer Followed by Surgery Study; dMMR, deficient mismatch repair; CT, computed tomography; DPYD, dihydropyrimidine dehydrogenase; EAC, esophageal adenocarcinoma; ESCC, esophageal squamous cell carcinoma; FLOT, fluorouracil, leucovorin, oxaliplatin, and docetaxel; FOLFOX, folinic acid, fluorouracil and oxaliplatin; FP, fluoropyrimidine; 5-FU, 5-fluorouracil; GC, gastric cancer; HER2, human epidermal growth factor receptor 2; IO, immunotherapy; MDT, multidisciplinary team; MSI, microsatellite instability; PD-L1, programmed cell death ligand 1; PET-CT, positron emission tomography-computed tomography; TPS, tumor proportion score.

**Figure 1** illustrates the steps for the diagnosis and treatment of upper GI cancers. Initial management should include either neoadjuvant chemoradiotherapy (CRT) with immunotherapy, perioperative chemotherapy (ChT), or surgery. Definitive CRT may be employed if the patient does not desire or is not eligible for surgery, whereas systemic therapy is required for unresectable disease. Systemic therapy includes ChT with a fluoropyrimidine and platinum; immune checkpoint inhibitors can extend survival (16, 17). Immunotherapy has been associated with improved quality of life (QoL) relative to ChT alone (5).

All individuals should be treated by expert multidisciplinary teams. It is essential to discuss patient preferences and goals early on (18). Individuals often have impaired QoL, and survivorship is a fundamental aspect of management (19, 20). Regular follow-up is required to monitor disease progression, and most patients require symptom management (21). Comprehensive supportive care is needed.

### 3.2 Best practices and rationale

#### 3.2.1 Diagnosis and workup

Clinicians should be aware of risk factors and screen patients with alarm symptoms. Early diagnosis is challenging but important to improve outcomes. There is no evidence to support screening for EC among patients presenting with gastroesophageal reflux disease (GERD) only (22). Patients with alarm symptoms such as dysphagia, odynophagia, anemia, weight loss, recurrent vomiting, loss of appetite, or GI bleeding should be evaluated. Risk factors for EAC include male sex, older age, White race, Barrett esophagus, nocturnal reflux, abdominal obesity, and tobacco use (23, 24). Risk factors for ESCC include low socioeconomic status, tobacco or alcohol use, consumption of hot or pickled foods, low consumption of fruits and vegetables, and radiation exposure (25).

The workup includes a complete blood count, metabolic panel, HbA1C, and EKG. Thorough staging is important to optimize therapy (26). PET-CT should be employed to confirm the diagnosis and the clinical stage, and to identify metastatic disease (27, 28); echo-endoscopy is another option (22, 29). For GC, a diagnostic laparoscopy may be part of the workup, and ferritin and vitamin B_12_ levels should be assessed. Clinicians should be aware of red flags for genetic contributions. If diffuse-type cancer is found, consider a detailed family history and genetic testing (30).

Patients should receive a baseline malnutrition assessment and follow-up. The Canadian Nutrition Screening Tool can identify patients who are at risk of malnutrition (31). The Canadian Malnutrition Task Force offers a toolkit and resources (32).

Reflex molecular testing is recommended. DPYD gene variant carriers are at risk for severe toxicity with ChT (33). Clinical judgment is required; most research has been carried out in Caucasians, and individuals of other ethnicities may need further testing (34–37). HER2 expression guides the selection of targeted therapy (trastuzumab), whereas dMMR/MSI and the PD-L1 combined positive score (CPS) or tumor proportion score (TPS) determine immunotherapy eligibility (38).

#### 3.2.2 Treatment of localized disease with curative intent

Esophagectomy and gastrectomy are the recommended modalities for patients with early-stage EC/GC and good performance status. Patients should undergo a perioperative assessment. Endoscopic submucosal dissection can be considered if the patient has the appropriate T stage, N0 disease, and is at high risk for open surgery complications. Upfront resection may be considered, but this can understage disease. Clinicians should assess whether the patient desires surgery. We recommend that care partners be included in all treatment decisions.

Multidisciplinary team (MDT) discussions that include a thoracic surgeon should be held for all patients. MDT discussion is essential, especially if the cancer is upstaged after PET-CT. Clinicians should consider whether research protocols are available.

Patients should receive neoadjuvant CRT with immunotherapy, or perioperative ChT. Curative-intent strategies include CROSS for ESCC (carboplatin + paclitaxel with radiotherapy), and perioperative FLOT (5-fluorouracil, leucovorin, oxaliplatin, and docetaxel) for EAC/GEJC/GC (39, 40). Other ChT regimens for ESCC include FOLFOX (folinic acid, fluorouracil, and oxaliplatin), carboplatin/etoposide, and carboplatin/paclitaxel. CROSS is an option for EAC/GEJC, but as shown by the ESOPEC trial, FLOT is preferred based on longer overall survival (OS) (41). Treatment decisions should be made in a multidisciplinary forum.

For GC, ChT options include perioperative FLOT, a fluoropyrimidine + cisplatin/oxaliplatin, and an adjuvant fluoropyrimidine with oxaliplatin or docetaxel (if there was no preoperative ChT). In the curative setting, there is currently no role for HER2-directed therapy. Clinicians should refer to provincial or international guidelines for further details of regimens (11–14, 17, 29, 42, 43)

The addition of immunotherapy to neoadjuvant or perioperative ChT is still experimental (2). Perioperative durvalumab + FLOT improved the rate of pathological complete response (pCR) in patients with GC/GEJC, compared with placebo + FLOT, in MATTERHORN (44). In KEYNOTE-585, neoadjuvant and adjuvant pembrolizumab added to ChT improved pCR among participants with resectable GC/GEJC (45). For MSI-H/dMMR GC/GEJC, trials suggest an important role of neoadjuvant immunotherapy (46). The NEONIPIGA trial reported 58.6% pCR after neoadjuvant nivolumab + ipilimumab, and the INFINITY trial demonstrated 60% pCR among patients who received neoadjuvant durvalumab + tremelimumab (47, 48). Patients with resectable MSI-H/dMMR adenocarcinoma should be included in a clinical trial or offered immunotherapy, if available.

Antibiotics should be prescribed carefully. Antibiotics within 1-2 months of initiating immunotherapy have been linked to poor survival (49–51). Clinicians should carefully consider antibiotics and should not prescribe them reflexively.

Patients with EC/GEJC who received neoadjuvant CRT and have residual disease should receive adjuvant immunotherapy. Adjuvant nivolumab improves disease-free survival (DFS) among patients with residual EC/GEJC after surgery and neoadjuvant CRT (52). CheckMate 577 identified a 31% risk reduction for disease progression or death among patients who received one year of nivolumab vs placebo (53). Patients should be educated on recognizing immune-related AEs.

#### 3.2.3 Assessing patient and care partner preferences

The individual’s and care partner’s preferences should be discussed throughout the treatment journey. Many patients value autonomy in deciding whether to undergo surgery, choosing a systemic therapy, or changing therapy (18, 54–58). Integrating patient preferences into decision-making can improve compliance and increase the personalization of care (54, 59). Rather than facing difficult decisions alone, patients often wish to consider the needs of their loved ones and receive their support (60). Including care partners in decision-making enhances patients’ ability to process large amounts of new information and manage psychological distress (60, 61). Hence, both patients and care partners require reliable information and decision support (60, 62).

#### 3.2.4 Follow-up after surgery

Because of the complex nature of upper GI cancers, multipronged follow-up is essential. There is no standard, but a reasonable surveillance program would include a physical examination every 3-6 months for 1-2 years, then every 6-12 months for 3-5 years. For high-risk patients, CT of the chest and abdomen (with oral and IV contrast, unless contraindicated) should be considered every 6 months for the first 2 years and annually for up to 5 years (29). Surveillance endoscopy is controversial (63).

If the cancer recurs, restaging with CT is required. Local esophageal recurrence may require stenting or palliative radiation, but left laryngeal nerve involvement is a concern. Proximal tumors are difficult to stent due to their proximity to the upper esophageal sphincter.

#### 3.2.5 Treatment of unresectable/metastatic disease with life-prolonging intent

Immunotherapy should be incorporated into treatment for eligible individuals. The benefit of adding immunotherapy to ChT has been demonstrated in clinical trials, and efficacy correlates with PD-L1 expression. A general rule is as follows:

- For CPS <1, consider ChT alone
- For CPS 1-10, consider a longer course of ChT with or without immunotherapy
- For CPS ≥10, consider dropping ChT after prolonged response or toxicity and treating with immunotherapy alone or immunotherapy + 5-fluorouracil (5-FU)

For ESCC, the recommended ChT regimen is 5-FU + cisplatin/oxaliplatin. For EAC, the recommended regimens are FOLFOX/CAPOX, a fluoropyrimidine (5-FU or capecitabine) + oxaliplatin, or FLOT (rarely used for unresectable disease). For GC, a fluoropyrimidine and a platinum agent (oxaliplatin or cisplatin) are recommended.

The addition of immunotherapy to a ChT backbone benefits many patients. Generally, patients with CPS scores ≥10 should receive ChT with immunotherapy, whereas patients with CPS scores <1 (i.e., minimal PD-L1 expression) should receive ChT alone. For intermediate scores, consider combining ChT with pembrolizumab or nivolumab. Pembrolizumab yields the best results with CPS ≥10, whereas nivolumab yields the best results with TPS ≥1% (64, 65). For HER2-positive disease and CPS scores ≥1, trastuzumab should be added.

The benefit of immunotherapy for eso-gastric ACs has been demonstrated in several trials. CheckMate 649 identified better progression-free survival (PFS) and OS with nivolumab + ChT compared with placebo + ChT, among patients with EAC, GEJC, or GC (66). The benefit was greatest among patients with CPS ≥5 (66). Pembrolizumab + ChT led to improved survival among patients with GC/GEJC in KEYNOTE-859; individuals with CPS ≥10 experienced the greatest benefit (64). In KEYNOTE-811, which included patients with advanced or metastatic HER2-positive G/GEJ AC, pembrolizumab added to trastuzumab and ChT improved survival among individuals with CPS ≥1 (67).

For patients with advanced or metastatic MSI-H/dMMR EAC or G/GEJ AC, pembrolizumab or nivolumab should be used since these tumors are very sensitive to immunotherapy (4). The use of doublet immunotherapy, or combinations of immunotherapy and ChT, is still debated in this small patient population.

Immunotherapy with ChT is also beneficial for ESCC. In KEYNOTE-590, with a majority of ESCC patients, individuals who received pembrolizumab + ChT had a longer mOS than those who received ChT alone (68). Similarly, in CheckMate 648, an improved mOS was demonstrated with nivolumab + ChT vs placebo + ChT, with the greatest benefit in individuals with TPS ≥1% (65, 69). Other agents, such as camrelizumab, toripalimab, and sintilimab, have also demonstrated survival improvements (70–72). At present, these agents are not available in Canada.

According to a meta-analysis of randomized controlled trials, immunotherapy improves health-related quality of life (HRQoL) relative to ChT among individuals with advanced eso-gastric cancer (5). Immunotherapy is also associated with better control of pain and dysphagia than ChT alone; these findings were attributed to lower levels of inflammation and cytotoxic effects (5).

Nivolumab + ChT is Health Canada indicated for HER2-negative advanced/metastatic GC, GEJC, and esophageal AC, regardless of PD-L1; and for unresectable or metastatic ESCC with tumor cell PD-L1 expression ≥1% (52). Nivolumab + ipilimumab is also indicated for unresectable or metastatic ESCC with PD-L1 expression ≥1% (52), but is not available in Canada. Pembrolizumab + ChT is indicated for HER2-negative unresectable/metastatic EC (regardless of PD-L1 or histology), for HER2-negative unresectable/metastatic G/GEJ AC, regardless of PD-L1; and for HER2-positive, unresectable/metastatic G/GEJ AC with CPS ≥1, with trastuzumab (73).

Immunotherapy offers significant benefits, but access is province dependent. Clinicians are encouraged to connect patients with support services, financial navigation, and access programs (74).

#### 3.2.6 Survivorship issues

##### 3.2.6.1 Nutrition

All patients with upper GI cancer should undergo nutrition assessments starting early in the disease course. We recommend involving a dietitian to address challenges such as weight loss, protein deficiency, and aspiration. EC and GC are associated with malnutrition, which negatively affects treatment outcomes and QoL (21, 75–78). Individuals who are at moderate to high risk of malnutrition are almost five times more likely to experience postoperative complications compared with those who are not at risk (78). In a study of individuals with ESCC treated at Princess Margaret Cancer Centre, 58% were malnourished, and these individuals had poor survival (75). Similar trends have been identified in GC (76, 77). In particular, iron deficiency, vitamin B_12_ deficiency, and dumping syndrome are common in gastrectomy patients (79).

The use of validated screening tools has been linked to reduced rates of malnutrition (80). In individuals with GC, nutrition support is associated with better outcomes after ChT and surgery (81). Enteral immunonutrition reduces the incidence of infectious complications after esophagectomy, compared with standard enteral nutrition (82). Clinicians should refer to the ESPEN guidelines (83) for the details of nutrition regimens.

##### 3.2.6.2 Dysphagia/reflux management

We suggest discussing patient preferences for dysphagia management. Dysphagia scoring may be useful to monitor symptoms (84, 85). Management depends on the tumor length, location, and residual luminal diameter. Radiation, ChT, and/or palliative stenting may be employed, as well as a feeding tube if necessary. The decision to use radiation may depend on how much benefit the patient experiences from ChT; radiation may be delayed for later use as a salvage therapy. A thoracic surgeon or gastroenterologist should review the case for a repeat endoscopy and possible stent. Intermittent balloon dilatation is helpful for short lesions, especially in the proximal esophagus.

##### 3.2.6.3 Psychosocial support

Patients should be referred to support groups such as My Gut Feeling (https://www.mygutfeeling.ca/) at an early stage, and clinicians should access the Canadian Association of Psychosocial Oncology (CAPO) to find practitioners with expertise in supporting cancer patients. Individuals with upper GI cancer have high levels of stress, anxiety, and depression (86). Psychosocial support should be offered throughout the disease course (86–88). Governments and organizations such as the Canadian Cancer Society, BC Cancer, Alberta Health Services, and the Government of Québec, offer support and counseling. Individuals may be very reliant on their care partner or support network; care partners also have significant psychosocial needs.

##### 3.2.6.4 Other supports

We suggest involving palliative care early in treatment so that individuals can receive support to improve their QoL, refine their treatment goals, and access pain management. Individuals are often more accepting of targeted therapy than ChT, but delaying ChT may render them ineligible for further treatment. Clinicians should ensure that the patient and care partner understand the implications of each option. An end-of-life discussion should be held to clarify the level of care and prognosis. Early palliative care is associated with a lower rate of ED visits and hospitalizations in the last month of life (89, 90).

## 4 Discussion

It is well established that the addition of immunotherapy to ChT is beneficial for patients with advanced or metastatic disease. The place of immunotherapy in neoadjuvant and perioperative approaches is still being investigated; nonetheless, there is a clear benefit of adjuvant therapy for patients with EC/GEJC who have residual disease after chemoradiation and surgery. Therefore, we recommend incorporating immunotherapy into treatment strategies where appropriate. It is essential to assess patient and care partner goals for treatment on an ongoing basis.

Ongoing trials such as DESTINY-Gastric03 (trastuzumab deruxtecan in HER2-positive GC) and SKYSCRAPER-07 (tiragolumab + atezolizumab in ESCC) will provide further insights as to the roles of targeted agents and immunotherapy (91, 92). Recent studies using zolbetuximab, an antibody directed against claudin 18.2, showed extended OS among patients with advanced GC/GEJC (93, 94). The eventual addition of zolbetuximab to the therapeutic arsenal will provide more options, especially for low-CPS tumors. Research is also providing insights to improve supportive care, as in a trial which demonstrated improvement of cachexia through inhibition of the growth differentiation factor 15 (GDF-15) (95).

PD-L1 expression has an important role in therapy selection, although some aspects (e.g., cases with low to intermediate PD-L1 expression) remain controversial (96). Clinicians should use their judgment in applying this biomarker in the context of the patient’s history and disease course. Other biomarkers, such as DPYD, MSI/dMMR, and HER2, are also essential in individualizing therapy. Unfortunately, funding for molecular testing is not always available, and access is province dependent (97). Clinicians are encouraged to advocate for reflex testing and to offer access to clinical trials and experimental protocols.

The algorithm presented here is based on high-quality evidence and experience with the management of upper GI cancers. We envision this algorithm being used by any practitioner who manages upper GI cancers in Canada to inform clinical decisions. This algorithm aligns with recent international guidelines, and we refer clinicians to other documents for the details of later-line regimens (17, 29, 43).

### 4.1 Strengths and limitations

Strengths of this study include that the study addressed current areas of educational need in the field of upper GI cancer treatment, based on responses to a needs assessment survey. A novel approach was employed, consisting of asynchronous discussion of upper GI cancer cases, overseen by an expert Scientific Planning Committee of experts with diverse backgrounds. The algorithm includes perspectives provided by a patient representative. The best practices reported are based on the experiences of clinicians practicing across Canada in multiple specialties, and most best practices are supported by recent randomized controlled trials. Limitations include that some best practices may be region dependent; applicability will depend on local resources, access, and insurance.

## Data Availability

The raw data supporting the conclusions of this article will be made available by the authors, without undue reservation.

## Glossary

AC: adenocarcinoma
ASCO: American Society of Clinical Oncology
CAPO: Canadian Association of Psychosocial Oncology
ChT: chemotherapy
CEA: carcinoembryonic antigen
CPS: PD-L1 expression combined positive score
CROSS: chemoradiotherapy for esophageal cancer followed by surgery study
CRT: chemoradiation therapy
dMMR: deficient mismatch repair
DPYD: dihydropyrimidine dehydrogenase
EAC: esophageal adenocarcinoma
EC: esophageal cancer
ESCC: esophageal squamous cell carcinoma
ESD: endoscopic submucosal dissection
ESMO: European Society for Medical Oncology
ESPEN: European Society for Clinical Nutrition and Metabolism
FLOT: 5-fluorouracil, leucovorin (folinic acid), oxaliplatin, and docetaxel
FOLFOX: folinic acid, 5-fluorouracil, and oxaliplatin
5-FU: 5-fluorouracil
GC: gastric cancer
GEJC: gastroesophageal junction cancer
HR: hazard ratio
HER2: human epidermal growth factor receptor 2
MDT: multidisciplinary team
mOS: median overall survival
mPFS: median progression-free survival
MSI: microsatellite instability
NCCN: National Comprehensive Cancer Network
pCR: pathological complete response
PD-L1: programmed cell death ligand-1
PEG: percutaneous endoscopic gastrostomy
PET-CT: positron emission tomography-computed tomography
TPS: tumor proportion score

## References

[1] Puhr HC Prager GW Ilhan-Mutlu A. How we treat esophageal squamous cell carcinoma. ESMO Open. (2023) 8:100789. doi: 10.1016/j.esmoop.2023.100789 36791637 PMC9958251

[2] Dennehy C Khan AF Zaidi AH Lam VK. The evolving landscape of neoadjuvant immunotherapy in gastroesophageal cancer. Cancers (Basel). (2024) 16:286. doi: 10.3390/cancers16020286 38254776 PMC10814157

[3] Le DT Ott PA Korytowsky B Le H Le TK Zhang Y. Real-world treatment patterns and clinical outcomes across lines of therapy in patients with advanced/metastatic gastric or gastroesophageal junction cancer. Clin Colorectal Cancer. (2020) 19:32–8.e3. doi: 10.1016/j.clcc.2019.09.001 31813769

[4] Dedecker H Teuwen L-A Vandamme T Domen A Prenen H. The role of immunotherapy in esophageal and gastric cancer. Clin Colorectal Cancer. (2023) 22:175–82. doi: 10.1016/j.clcc.2023.03.001 37005190

[5] Gupta K Roy AM Attwood K Nipp RD Mukherjee S. Effects of immunotherapy on quality-of-life outcomes in patients with gastroesophageal cancers: A meta-analysis of randomized controlled trials. Healthcare (Basel). (2024) 12:1496. doi: 10.3390/healthcare12151496 39120199 PMC11311609

[6] Narita Y Muro K. Updated immunotherapy for gastric cancer. J Clin Med. (2023) 12:2636. doi: 10.3390/jcm12072636 37048719 PMC10094960

[7] Kwak HV Banks KC Hung YY Alcasid NJ Susai CJ Patel A. Adjuvant immunotherapy in curative intent esophageal cancer resection patients: Real-world experience within an integrated health system. Cancers (Basel). (2023) 15:5317. doi: 10.3390/cancers15225317 38001577 PMC10669669

[8] Habbous S Yermakhanova O Forster K Holloway CMB Darling G. Variation in diagnosis, treatment, and outcome of esophageal cancer in a regionalized care system in Ontario, Canada. JAMA Netw Open. (2021) 4:e2126090–e. doi: 10.1001/jamanetworkopen.2021.26090 PMC8456383 34546371

[9] Schendel J Jost E Mah M Mack L McCall M Gu N. Gastric cancer management in elderly patients: A population-based study of treatment patterns and outcomes in gastric cancer patients ≥ 75 years from Alberta, Canada. Am J Surg. (2021) 221:839–43. doi: 10.1016/j.amjsurg.2020.03.006 32222273

[10] Merchant SJ Kong W Gyawali B Hanna TP Chung W Nanji S. First-line palliative chemotherapy for esophageal and gastric cancer: Practice patterns and outcomes in the general population. JCO Oncol Pract. (2021) 17:e1537–e50. doi: 10.1200/OP.20.00397 33449833

[11] Cancer Care Ontario. Esophageal cancer pathway map. Available online at: https://www.cancercareontario.ca/en/pathway-maps/esophageal-cancer (Accessed July 28, 2024).

[12] Cancer Care Alberta Guideline Resource Unit. Clinical practice guideline GI-009 – version 6. Available online at: https://www.albertahealthservices.ca/assets/info/hp/cancer/if-hp-cancer-guide-gi008-gastric.pdf (Accessed July 28, 2024).

[13] BC Cancer Agency. Family Practice Oncology Network Clinical Practice Guidelines. Available online at: http://www.bccancer.bc.ca/health-professionals/networks/family-practice-oncology-network/guidelines-protocols (Accessed July 28, 2024).

[14] Cancer Care Manitoba. Practice guideline: Disease management guideline for the curative treatment of gastric cancer. Available online at: https://www.cancercare.mb.ca/export/sites/default/For-Health-Professionals/.galleries/files/treatment-guidelines-rro-files/practice-guidelines/gastro-intestinal/Guideline-for-the-curative-treatment-of-gastric-cancer-CCMB_9.pdf (Accessed July 28, 2024).

[15] Upper GI cancer masterclass. Available online at: https://dxlink.ca/programs/GI-Cancer-Masterclass/Home (Accessed March 6, 2025).

[16] Svrcek M Voron T André T Smyth EC de la Fouchardière C. Improving individualised therapies in localised gastro-oesophageal adenocarcinoma. Lancet Oncol. (2024) 25:e452–e63. doi: 10.1016/S1470-2045(24)00180-3 39214116

[17] Shah MA Kennedy EB Alarcon-Rozas AE Alcindor T Bartley AN Malowany AB. Immunotherapy and targeted therapy for advanced gastroesophageal cancer: ASCO guideline. J Clin Oncol. (2023) 41:1470–91. doi: 10.1200/JCO.22.02331 36603169

[18] Larsen MK Schultz H Mortensen MB Birkelund R. Patients’ experiences with illness, treatment, and decision-making for esophageal cancer: A qualitative study in a Danish hospital setting. Glob Qual Nurs Res. (2020) 7:2333393620935098. doi: 10.1177/2333393620935098 32656299 PMC7328478

[19] Bennett AE O’Neill L Connolly D Guinan E Boland L Doyle S. Perspectives of esophageal cancer survivors on diagnosis, treatment, and recovery. Cancers (Basel). (2020) 13:100. doi: 10.3390/cancers13010100 33396253 PMC7796170

[20] Graham L Wikman A. Toward improved survivorship: Supportive care needs of esophageal cancer patients, a literature review. Dis Esophagus. (2016) 29:1081–9. doi: 10.1111/dote.2016.29.issue-8 26455727

[21] Jeon M Jang H Jeon H Park CG Kim S. Long-term late effects in older gastric cancer survivors: Survival analysis using Cox hazard regression model by retrospective electronic health records. Support Care Cancer. (2023) 32:29. doi: 10.1007/s00520-023-08202-7 38099981 PMC10724335

[22] Hamel C Ahmadzai N Beck A Thuku M Skidmore B Pussegoda K. Screening for esophageal adenocarcinoma and precancerous conditions (dysplasia and Barrett’s esophagus) in patients with chronic gastroesophageal reflux disease with or without other risk factors: Two systematic reviews and one overview of reviews to inform a guideline of the Canadian Task Force on Preventive Health Care (CTFPHC). Syst Rev. (2020) 9:20. doi: 10.1186/s13643-020-1275-2 31996261 PMC6990541

[23] Groulx S Limburg H Doull M Klarenbach S Singh H Wilson BJ. Guideline on screening for esophageal adenocarcinoma in patients with chronic gastroesophageal reflux disease. CMAJ. (2020) 192:E768–e77. doi: 10.1503/cmaj.190814 PMC7828892 32631908

[24] Rubenstein JH Shaheen NJ. Epidemiology, diagnosis, and management of esophageal adenocarcinoma. Gastroenterology. (2015) 149:302–17.e1. doi: 10.1053/j.gastro.2015.04.053 25957861 PMC4516638

[25] Abnet CC Arnold M Wei WQ. Epidemiology of esophageal squamous cell carcinoma. Gastroenterology. (2018) 154:360–73. doi: 10.1053/j.gastro.2017.08.023 PMC5836473 28823862

[26] Deja A Włodarczyk M. Esophageal cancer - the utility of PET/CT in staging prior to chemoradiation. Rep Pract Oncol Radiother. (2023) 28:608–11. doi: 10.5603/rpor.96869 PMC10764044 38179288

[27] Bruzzi JF Munden RF Truong MT Marom EM Sabloff BS Gladish GW. PET/CT of esophageal cancer: Its role in clinical management. Radiographics. (2007) 27:1635–52. doi: 10.1148/rg.276065742 18025508

[28] Jayaprakasam VS Yeh R Ku GY Petkovska I III JLF Gollub M. Role of imaging in esophageal cancer management in 2020: Update for radiologists. Am J Roentgenol. (2020) 215:1072–84. doi: 10.2214/AJR.20.22791 32901568

[29] National Comprehensive Cancer Network. NCCN Clinical Practice Guidelines In Oncology (NCCN Guidelines). Version 4.2024. Available online at: https://www.nccn.org/guidelines/nccn-guidelines/guidelines-detail?category=1&id=1433 (Accessed May 15, 2024).

[30] Brooks-Wilson AR Kaurah P Suriano G Leach S Senz J Grehan N. Germline E-cadherin mutations in hereditary diffuse gastric cancer: Assessment of 42 new families and review of genetic screening criteria. J Med Genet. (2004) 41:508–17. doi: 10.1136/jmg.2004.018275 PMC1735838 15235021

[31] Canadian Malnutrition Task Force. Nutrition screening. Available online at: https://nutritioncareinCanada.ca/resources-and-tools/hospital-care-inpac/screening (Accessed May 15, 2024).

[32] Canadian Malnutrition Task Force. Hospital care/INPAC. Available online at: https://nutritioncareinCanada.ca/resources-and-tools/hospital-care-inpac/overview (Accessed August 26, 2024).

[33] Jolivet C Nassabein R Soulières D Weng X Amireault C Ayoub JP. Implementing DPYD*2a genotyping in clinical practice: The Quebec, Canada, experience. Oncologist. (2021) 26:e597–602. doi: 10.1002/onco.13626 PMC8018309 33274825

[34] Cancer Care Ontario. Fluoropyrimidine treatment in patients with dihydropyrimidine dehydrogenase (DPD) deficiency: Guidance for clinicians. Available online at: https://www.cancercareontario.ca/en/guidelines-advice/types-of-cancer/73951 (Accessed July 28, 2024).

[35] Pratt VM Cavallari LH Fulmer ML Gaedigk A HaChad H Ji Y. DPYD genotyping recommendations: A joint consensus recommendation of the Association for Molecular Pathology, American College of Medical Genetics and Genomics, Clinical Pharmacogenetics Implementation Consortium, College of American Pathologists, Dutch Pharmacogenetics Working Group of the Royal Dutch Pharmacists Association, European Society for Pharmacogenomics and Personalized Therapy, Pharmacogenomics Knowledgebase, and Pharmacogene Variation Consortium. J Mol Diagn. (2024) 26(10):851–63. doi: 10.1016/j.jmoldx.2024.05.015 PMC12813241 39032821

[36] White C Scott RJ Paul C Ziolkowski A Mossman D Ackland S. Ethnic diversity of DPD activity and the DPYD gene: Review of the literature. Pharmgenomics Pers Med. (2021) 14:1603–17. doi: 10.2147/PGPM.S337147 PMC8668257 34916829

[37] Chan TH Zhang JE Pirmohamed M. DPYD genetic polymorphisms in non-European patients with severe fluoropyrimidine-related toxicity: A systematic review. Br J Cancer. (2024) 131:498–514. doi: 10.1038/s41416-024-02754-z 38886557 PMC11300675

[38] Rai V Abdo J Agrawal DK. Biomarkers for early detection, prognosis, and therapeutics of esophageal cancers. Int J Mol Sci. (2023) 24:3316. doi: 10.3390/ijms24043316 36834728 PMC9968115

[39] Lorenzen S Pauligk C Homann N Schmalenberg H Jäger E Al-Batran SE. Feasibility of perioperative chemotherapy with infusional 5-FU, leucovorin, and oxaliplatin with (FLOT) or without (FLO) docetaxel in elderly patients with locally advanced esophagogastric cancer. Br J Cancer. (2013) 108:519–26. doi: 10.1038/bjc.2012.588 PMC3593547 23322206

[40] Homann N Pauligk C Luley K Werner Kraus T Bruch HP Atmaca A. Pathological complete remission in patients with oesophagogastric cancer receiving preoperative 5-fluorouracil, oxaliplatin and docetaxel. Int J Cancer. (2012) 130:1706–13. doi: 10.1002/ijc.v130.7 21618509

[41] Hoeppner J Brunner T Lordick F Schmoor C Kulemann B Neumann UP. Prospective randomized multicenter phase III trial comparing perioperative chemotherapy (FLOT protocol) to neoadjuvant chemoradiation (CROSS protocol) in patients with adenocarcinoma of the esophagus (ESOPEC trial). J Clin Oncol. (2024) 42:LBA1. doi: 10.1200/JCO.2024.42.17_suppl.LBA1 PMC4952147 27435280

[42] Lordick F Candia Montero L Castelo-Branco L Pentheroudakis G Sessa C Smyth E. ESMO gastric cancer living guideline, v1.2. Available online at: https://www.esmo.org/living-guidelines/esmo-gastric-cancer-living-guideline (Accessed November 15, 2023).

[43] Lordick F Carneiro F Cascinu S Fleitas T Haustermans K Piessen G. Gastric cancer: ESMO clinical practice guideline for diagnosis, treatment and follow-up. Ann Oncol. (2022) 33:1005–20. doi: 10.1016/j.annonc.2022.07.004 35914639

[44] Janjigian YY Van Cutsem E Muro K Wainberg Z Al-Batran SE Hyung WJ. MATTERHORN: Phase III study of durvalumab plus FLOT chemotherapy in resectable gastric/gastroesophageal junction cancer. Future Oncol. (2022) 18:2465–73. doi: 10.2217/fon-2022-0093 35535555

[45] Shitara K Rha SY Wyrwicz LS Oshima T Karaseva N Osipov M. Neoadjuvant and adjuvant pembrolizumab plus chemotherapy in locally advanced gastric or gastro-oesophageal cancer (KEYNOTE-585): An interim analysis of the multicentre, double-blind, randomised phase 3 study. Lancet Oncol. (2024) 25:212–24. doi: 10.1016/S1470-2045(23)00541-7 38134948

[46] Wu D Yang L Yan Y Jiang Z Liu Y Dong P. Neoadjuvant immunotherapy improves outcomes for resectable gastroesophageal junction cancer: A systematic review and meta-analysis. Cancer Med. (2024) 13:e7176. doi: 10.1002/cam4.v13.9 38716645 PMC11077431

[47] André T Tougeron D Piessen G de la Fouchardière C Louvet C Adenis A. Neoadjuvant nivolumab plus ipilimumab and adjuvant nivolumab in localized deficient mismatch repair/microsatellite instability-high gastric or esophagogastric junction adenocarcinoma: The GERCOR NEONIPIGA phase II study. J Clin Oncol. (2023) 41:255–65. doi: 10.1200/JCO.22.006 PMC9839243 35969830

[48] Pietrantonio F Raimondi A Lonardi S Murgioni S Cardellino GG Tamberi S. INFINITY: A multicentre, single-arm, multi-cohort, phase II trial of tremelimumab and durvalumab as neoadjuvant treatment of patients with microsatellite instability-high (MSI) resectable gastric or gastroesophageal junction adenocarcinoma (GAC/GEJAC). J Clin Oncol. (2023) 41:358–. doi: 10.1200/JCO.2023.41.4_suppl.358

[49] Zhou J Huang G Wong WC Hu DH Zhu JW Li R. The impact of antibiotic use on clinical features and survival outcomes of cancer patients treated with immune checkpoint inhibitors. Front Immunol. (2022) 13:968729. doi: 10.3389/fimmu.2022.968729 35967438 PMC9367677

[50] He J Li H Jia J Liu Y Zhang N Wang R. Mechanisms by which the intestinal microbiota affects gastrointestinal tumours and therapeutic effects. Mol BioMed. (2023) 4:45. doi: 10.1186/s43556-023-00157-9 38032415 PMC10689341

[51] Huang XZ Gao P Song YX Xu Y Sun JX Chen XW. Antibiotic use and the efficacy of immune checkpoint inhibitors in cancer patients: A pooled analysis of 2740 cancer patients. Oncoimmunology. (2019) 8:e1665973. doi: 10.1080/2162402X.2019.1665973 31741763 PMC6844307

[52] Opdivo (nivolumab) product monograph. Montreal, Canada: Bristol-Myers Squibb Canada (2024).

[53] Kelly RJ Ajani JA Kuzdzal J Zander T Van Cutsem E Piessen G. Adjuvant nivolumab in resected esophageal or gastroesophageal junction cancer. N Engl J Med. (2021) 384:1191–203. doi: 10.1056/NEJMoa2032125 33789008

[54] Hermus M van der Wilk BJ Chang R Dekker JWT Coene PLO Nieuwenhuijzen GAP. Esophageal cancer patients’ need for information and support in making a treatment decision between standard surgery and active surveillance. Cancer Med. (2023) 12:17266–72. doi: 10.1002/cam4.v12.16 PMC10501224 37392175

[55] Czornik M Weis J Kiemen A Schmoor C Hipp J Hoeppner J. Needs, preferences, and patient participation for a randomized controlled trial on postneoadjuvant complete tumor response: A qualitative study of patients with esophageal cancer. Support Care Cancer. (2024) 32:650. doi: 10.1007/s00520-024-08845-0 39256205 PMC11387432

[56] de Bekker-Grob EW Niers EJ van Lanschot JJ Steyerberg EW Wijnhoven BP. Patients’ preferences for surgical management of esophageal cancer: A discrete choice experiment. World J Surg. (2015) 39:2492–9. doi: 10.1007/s00268-015-3148-8 PMC4554743 26170156

[57] Noordman BJ de Bekker-Grob EW Coene PPLO van der Harst E Lagarde SM Shapiro J. Patients’ preferences for treatment after neoadjuvant chemoradiotherapy for oesophageal cancer. Br J Surg. (2018) 105:1630–8. doi: 10.1002/bjs.10897 29947418

[58] Nizet P Grivel C Rabeau P Pecout S Evin A Labarthe SP. Patients’ preferences in therapeutic decision-making in digestive oncology: A single centre cross-sectional observational study. Sci Rep. (2023) 13:8534. doi: 10.1038/s41598-023-35407-x 37237043 PMC10220004

[59] Yeo HY Liew AC Chan SJ Anwar M Han CH-W Marra CA. Understanding patient preferences regarding the important determinants of breast cancer treatment: A narrative scoping review. Patient Prefer Adherence. (2023) 17:2679–706. doi: 10.2147/PPA.S432821 PMC10625390 37927344

[60] Hermus M van der Sluis PC Wijnhoven BPL van der Zijden CJ van Busschbach JJ Lagarde SM. Decision-making experiences of patients and partners opting for active surveillance in esophageal cancer treatment. Patient Educ Couns. (2024) 127:108361. doi: 10.1016/j.pec.2024.108361 38936160

[61] Cincidda C Pizzoli SF Ongaro G Oliveri S Pravettoni G. Caregiving and shared decision making in breast and prostate cancer patients: A systematic review. Curr Oncol. (2023) 30:803–23. doi: 10.3390/curroncol30010061 PMC9857468 36661710

[62] McNair AGK MacKichan F Donovan JL Brookes ST Avery KNL Griffin SM. What surgeons tell patients and what patients want to know before major cancer surgery: A qualitative study. BMC Cancer. (2016) 16:258. doi: 10.1186/s12885-016-2292-3 27036216 PMC4815149

[63] Haveman JW. Intensive surveillance after esophagectomy in patients with esophageal cancer: When, why, and how often? Ann Surg Oncol. (2023) 30:1948–9. doi: 10.1245/s10434-022-12767-8 36372848

[64] Rha SY Oh DY Yañez P Bai Y Ryu MH Lee J. Pembrolizumab plus chemotherapy versus placebo plus chemotherapy for HER2-negative advanced gastric cancer (KEYNOTE-859): A multicentre, randomised, double-blind, phase 3 trial. Lancet Oncol. (2023) 24:1181–95. doi: 10.1016/S1470-2045(23)00515-6 37875143

[65] Doki Y Ajani JA Kato K Xu J Wyrwicz L Motoyama S. Nivolumab combination therapy in advanced esophageal squamous-cell carcinoma. N Engl J Med. (2022) 386:449–62. doi: 10.1056/NEJMoa2111380 35108470

[66] Shitara K Moehler MH Ajani JA Shen L Garrido M Gallardo C. Nivolumab (nivo) + chemotherapy (chemo) vs chemo as first-line (1L) treatment for advanced gastric cancer/gastroesophageal junction cancer/esophageal adenocarcinoma (GC/GEJC/EAC): 4 year (yr) follow-up of CheckMate 649. J Clin Oncol. (2024) 42:306. doi: 10.1200/JCO.2024.42.3_suppl.306 PMC11185916 38382001

[67] Janjigian YY Kawazoe A Bai Y Xu J Lonardi S Metges JP. Pembrolizumab plus trastuzumab and chemotherapy for HER2-positive gastric or gastro-oesophageal junction adenocarcinoma: Interim analyses from the phase 3 KEYNOTE-811 randomised placebo-controlled trial. Lancet. (2023) 402:2197–208. doi: 10.1016/S0140-6736(23)02033-0 37871604

[68] Metges J-P Kato K Sun J-M Shah MA Enzinger PC Adenis A. First-line pembrolizumab plus chemotherapy versus chemotherapy in advanced esophageal cancer: Longer-term efficacy, safety, and quality-of-life results from the phase 3 KEYNOTE-590 study. J Clin Oncol. (2022) 40:241. doi: 10.1200/JCO.2022.40.4_suppl.241

[69] Kato K Doki Y Chau I Xu J Wyrwicz L Motoyama S. Nivolumab plus chemotherapy or ipilimumab versus chemotherapy in patients with advanced esophageal squamous cell carcinoma (CheckMate 648): 29-month follow-up from a randomized, open-label, phase III trial. Cancer Med. (2024) 13:e7235. doi: 10.1002/cam4.v13.9 38716626 PMC11077338

[70] Luo H Lu J Bai Y Mao T Wang J Fan Q. Effect of camrelizumab vs placebo added to chemotherapy on survival and progression-free survival in patients with advanced or metastatic esophageal squamous cell carcinoma: The ESCORT-1st randomized clinical trial. JAMA. (2021) 326:916–25. doi: 10.1001/jama.2021.12836 PMC8441593 34519801

[71] Wang ZX Cui C Yao J Zhang Y Li M Feng J. Toripalimab plus chemotherapy in treatment-naïve, advanced esophageal squamous cell carcinoma (JUPITER-06): A multi-center phase 3 trial. Cancer Cell. (2022) 40:277–88. doi: 10.1016/j.ccell.2022.02.007 35245446

[72] Lu Z Wang J Shu Y Liu L Kong L Yang L. Sintilimab versus placebo in combination with chemotherapy as first line treatment for locally advanced or metastatic oesophageal squamous cell carcinoma (ORIENT-15): Multicentre, randomised, double blind, phase 3 trial. BMJ. (2022) 377:e068714. doi: 10.1136/bmj-2021-068714 35440464 PMC9016493

[73] Keytruda (pembrolizumab) product monograph. Kirkland, QC: Merck Canada Inc (2024).

[74] Wood TF Murphy RA. Tackling financial toxicity related to cancer care in Canada. CMAJ. (2024) 196:E297–E8. doi: 10.1503/cmaj.230677 PMC10927292 38467415

[75] Taylor K Espin-Garcia O Jiang DM Yokom D Ma LX Lim CH. Prognostic significance of malnutrition in metastatic esophageal squamous cell carcinoma. J Clin Oncol. (2019) 37:171–. doi: 10.1200/JCO.2019.37.4_suppl.171

[76] Guo ZQ Yu JM Li W Fu ZM Lin Y Shi YY. Survey and analysis of the nutritional status in hospitalized patients with Malignant gastric tumors and its influence on the quality of life. Support Care Cancer. (2020) 28:373–80. doi: 10.1007/s00520-019-04803-3 PMC6882767 31049672

[77] Nikniaz Z Somi MH Naghashi S. Malnutrition and weight loss as prognostic factors in the survival of patients with gastric cancer. Nutr Cancer. (2022) 74:3140–5. doi: 10.1080/01635581.2022.2059089 35373675

[78] Zhang J Xu W Zhang H Fan Y. Association between risk of malnutrition defined by patient-generated subjective global assessment and adverse outcomes in patients with cancer: A systematic review and meta-analysis. Public Health Nutr. (2024) 27:e105. doi: 10.1017/S1368980024000788 38533774 PMC11010050

[79] Teixeira Farinha H Bouriez D Grimaud T Rotariu AM Collet D Mantziari S. Gastro-intestinal disorders and micronutrient deficiencies following oncologic esophagectomy and gastrectomy. Cancers (Basel). (2023) 15:3554. doi: 10.3390/cancers15143554 37509216 PMC10376982

[80] Eglseer D Halfens RJ Lohrmann C. Is the presence of a validated malnutrition screening tool associated with better nutritional care in hospitalized patients? Nutrition. (2017) 37:104–11. doi: 10.1016/j.nut.2016.12.016 28359355

[81] Triantafillidis JK Papakontantinou J Antonakis P Konstadoulakis MM Papalois AE. Enteral nutrition in operated-on gastric cancer patients: An update. Nutrients. (2024) 16:1639. doi: 10.3390/nu16111639 38892572 PMC11174039

[82] Tian X Jin YF Liu XL Chen H Chen WQ Jiménez-Herrera MF. Network meta-analysis of the optimal time of applying enteral immunonutrition in esophageal cancer patients receiving esophagectomy. Support Care Cancer. (2022) 30:7133–46. doi: 10.1007/s00520-022-07058-7 35445866

[83] Muscaritoli M Arends J Bachmann P Baracos V Barthelemy N Bertz H. ESPEN practical guideline: Clinical nutrition in cancer. Clin Nutr. (2021) 40:2898–913. doi: 10.1016/j.clnu.2021.02.005 33946039

[84] Mellow MH Pinkas H. Endoscopic laser therapy for Malignancies affecting the esophagus and gastroesophageal junction. Analysis of technical and functional efficacy. Arch Intern Med. (1985) 145:1443–6. doi: 10.1001/archinte.1985.00360080117017 4026476

[85] Ripley RT Sarkaria IS Grosser R Sima CS Bains MS Jones DR. Pretreatment dysphagia in esophageal cancer patients may eliminate the need for staging by endoscopic ultrasonography. Ann Thorac Surg. (2016) 101:226–30. doi: 10.1016/j.athoracsur.2015.06.062 PMC4765728 26603024

[86] Pinto E Cavallin F Scarpa M. Psychological support of esophageal cancer patient? J Thorac Dis. (2019) 11:S654–s62. doi: 10.21037/jtd.2019.02.34 PMC6503274 31080642

[87] Howell D Mayo S Currie S Jones G Boyle M Hack T. Psychosocial health care needs assessment of adult cancer patients: A consensus-based guideline. Support Care Cancer. (2012) 20:3343–54. doi: 10.1007/s00520-012-1468-x 22581015

[88] Li ZY Ren JY Zhong JD Zhang JE. Understanding the supportive care needs among discharged patients with esophageal cancer after esophagectomy: A qualitative study. Eur J Oncol Nurs. (2023) 64:102337. doi: 10.1016/j.ejon.2023.102337 37290163

[89] Kitti PM Anttonen AM Leskelä R-L Saarto T. End-of-life care of patients with esophageal or gastric cancer: Decision making and the goal of care. Acta Oncol. (2022) 61:1173–8. doi: 10.1080/0284186X.2022.2114379 36005550

[90] Klastersky J Libert I Libert Y Echterbille M-A. A new comprehensive and stratified concept for supportive care in cancer patients. Curr Opin Oncol. (2024) 36:206–10. doi: 10.1097/CCO.0000000000001039 38726807

[91] Goodman KA Xu RH Chau I Chen MH Cho BC Shah MA. SKYSCRAPER-07: A phase III, randomized, double-blind, placebo-controlled study of atezolizumab with or without tiragolumab in patients with unresectable ESCC who have not progressed following definitive concurrent chemoradiotherapy. J Clin Oncol. (2022) 40:TPS374. doi: 10.1200/JCO.2022.40.4_suppl.TPS374

[92] Janjigian YY Raoufmoghaddam S Sztachelska M Winter M Das S. Phase 1b/2, open-label dose-escalation and -expansion study evaluating trastuzumab deruxtecan (T-DXd) monotherapy and combinations in patients (pts) with HER2+ and HER2-low gastric cancer (GC): DESTINY-Gastric03 (DG-03). J Clin Oncol. (2024) 42:TPS424. doi: 10.1200/JCO.2024.42.3_suppl.TPS424

[93] Shah MA Shitara K Ajani JA Bang Y-J Enzinger P Ilson D. Zolbetuximab plus CAPOX in CLDN18.2-positive gastric or gastroesophageal junction adenocarcinoma: The randomized, phase 3 GLOW trial. Nat Med. (2023) 29:2133–41. doi: 10.1038/s41591-023-02465-7 PMC10427418 37524953

[94] Shitara K Lordick F Bang Y-J Enzinger P Ilson D Shah MA. Zolbetuximab plus mFOLFOX6 in patients with CLDN18.2-positive, HER2-negative, untreated, locally advanced unresectable or metastatic gastric or gastro-oesophageal junction adenocarcinoma (SPOTLIGHT): A multicentre, randomised, double-blind, phase 3 trial. Lancet. (2023) 401:1655–68. doi: 10.1016/S0140-6736(23)00620-7 37068504

[95] Groarke JD Crawford J Collins SM Lubaczewski S Roeland EJ Naito T. Ponsegromab for the treatment of cancer cachexia. N Engl J Med. (2024) 391:2291–303. doi: 10.1056/NEJMoa2409515 39282907

[96] Kim HD Shin J Song IH Hyung J Lee H Ryu MH. Discordant PD-L1 results between 28-8 and 22c3 assays are associated with outcomes of gastric cancer patients treated with nivolumab plus chemotherapy. Gastric Cancer. (2024) 27:819–26. doi: 10.1007/s10120-024-01500-x 38647978

[97] Snow S Brezden-Masley C Carter MD Dhani N Macaulay C Ramjeesingh R. Barriers and unequal access to timely molecular testing results: Addressing the inequities in cancer care delays across Canada. Curr Oncol. (2024) 31:1359–75. doi: 10.3390/curroncol31030103 PMC10969404 38534936

